# Devaluation and sequential decisions: linking goal-directed and model-based behavior

**DOI:** 10.3389/fnhum.2014.00587

**Published:** 2014-08-04

**Authors:** Eva Friedel, Stefan P. Koch, Jean Wendt, Andreas Heinz, Lorenz Deserno, Florian Schlagenhauf

**Affiliations:** ^1^Department of Psychiatry and Psychotherapy, Charité – UniversitätsmedizinBerlin, Germany; ^2^Max Planck-Fellow Group “Cognitive and Affective Control of Behavioural Adaptation,” Max Planck Institute for Human Cognitive and Brain SciencesLeipzig, Germany

**Keywords:** model-based and model-free learning, habitual and goal-directed behavior, 2-step decision task, devaluation task, reinforcement learning, computational modeling

## Abstract

In experimental psychology different experiments have been developed to assess goal–directed as compared to habitual control over instrumental decisions. Similar to animal studies selective devaluation procedures have been used. More recently sequential decision-making tasks have been designed to assess the degree of goal-directed vs. habitual choice behavior in terms of an influential computational theory of model-based compared to model-free behavioral control. As recently suggested, different measurements are thought to reflect the same construct. Yet, there has been no attempt to directly assess the construct validity of these different measurements. In the present study, we used a devaluation paradigm and a sequential decision-making task to address this question of construct validity in a sample of 18 healthy male human participants. Correlational analysis revealed a positive association between model-based choices during sequential decisions and goal-directed behavior after devaluation suggesting a single framework underlying both operationalizations and speaking in favor of construct validity of both measurement approaches. Up to now, this has been merely assumed but never been directly tested in humans.

## Introduction

Habitual decisions arise from the retrospective, slow accumulation of rewards via iterative updating of expectations. In contrast, the goal-directed system prospectively considers future outcomes associated with an action. Thus, if outcome values change suddenly e.g., after devaluation (i.e., satiety), the goal-directed system enables quick behavioral adaptation, whereas the habitual system requires new reward experience before it can alter behavior accordingly (Balleine and Dickinson, 1998). Recently, this dual system theory has been advanced by the use of computational models of learning which either purely update reward expectations based on reward prediction errors (“model-free”) or aim to map possible actions to their potential outcomes (“model-based”; Daw et al., 2005). In their comprehensive review Dolan and Dayan (2013) subsume both concepts (goal-directed/habitual and model-based/model-free) under a single framework of reflective vs. reflexive decision making. Here, model-based choices are by definition goal-directed and model-free choices rest upon habitual learning. The authors provide a historical and conceptual framework for the evolution of dual systems theories with a reflexive and a reflective control system in cognitive neuroscience. This longstanding dichotomy has been described as goal-directed vs. habitual behavior by experimental psychologists while the model-free vs. model-based theory provides a computational account of the same construct.

Dolan and Dayan (2013) rank goal-directed behavior in humans in Generation 2, evolving from animal experiments in Generation 1. Generation 3 starts with the conceptual precision of goal-directed and habitual decision making as model-based vs. model-free learning on the basis of computational accounts in a reinforcement learning context. Even though both terminologies, goal-directed and model-based behavioral control, derive from the same framework, the different operationalizations have never been directly related in a human sample.

There are two main, but experimentally distinct, approaches to test the influence of both systems: outcome devaluation and sequential decision-making. First, devaluation paradigms require participants to overcome a previously trained action after outcome devaluation. Here, the goal-directed system adapts quickly based on an explicit action-outcome association. This is in sharp contrast to the habitual system that remains initially tied to the action acquired before devaluation because it relies on a stimulus-action association without direct representation of the link between action and a now devalued outcome. These paradigms have been developed in animal research (Dickinson, 1985) and were successfully translated to human research in healthy (Valentin et al., 2007; De Wit et al., 2009; Tricomi et al., 2009) and pathological conditions (De Wit et al., 2011; Gillan et al., 2011; Sjoerds et al., 2013). Second, sequential decision-making challenges an individual with a series of subsequent decisions to finally receive a reward (Generation 3). These tasks are characterized by a state-transition structure, which probabilistically determines the entered state after a given choice. Hence, a learner that acquires and uses this task structure (e.g., using a decision tree) by building and using an internal representation (a “model”) of the task is therefore labeled as “model-based.” This learner builds an internal representation of the task structure, which enables forward planning. Apparently, model-based learning is by definition goal-directed. A purely “model-free” learner neglects these transition schemes and simply repeats action sequences that were previously rewarded. Such tasks have been applied in healthy participants (Daw et al., 2011; Wunderlich et al., 2012; Smittenaar et al., 2013) and in one study in alcohol-dependent patients (Sebold et al., 2014). For both types of tasks, there is convincing evidence that human choices are influenced by both systems.

It is an on-going question whether these different measurements assess the same aspects of instrumental behavior (Doll et al., 2012; Dolan and Dayan, 2013). We assume that both measurements reflect the same construct and therefore shed light on similar mechanism from the perspectives of different experimental procedures that evolved from different fields (experimental psychology and computational theory). So far, this issue of construct validity has not been directly tested. However, the question of construct validity is important to address: in neuroscience research the two measurements have so far been treated almost equivalently and conclusions on presumably identical processes have been drawn in healthy human beings and also in severely ill individuals. Relating both measurements thus represents a coercive step to add to their conceptual precision.

To assess construct validity, we applied two tasks: a selective devaluation task (Valentin et al., 2007; Daw et al., 2011) and a sequential decision-making task (Daw et al., 2011) proven to capture the two constructs of goal-directed vs. habitual and model-based vs. model-free behavioral control separately using a within-subject design in 18 healthy male participants.

## Materials and methods

### Subjects

Eighteen right-handed healthy male subjects participated in the study. All participants were assessed for Axis I or II disorder with SCID-I Interview as well as for eating disorders with the Eating Attitude Test (EAT-26) (Garner et al., 1982) indicating no psychiatric or eating disorder in any of the subjects. Participants were pre-screened to ensure that they found tomato juice, chocolate milk and fruit tea pleasant and did not show any food intolerance or were not on a diet. All participants were asked to fast for at least 6 h before their scheduled arrival time, but were permitted and motivated to drink water before the experimental procedure. Upon arrival, participants rated their hunger on a visual analog scale (VAS) and informed the instructor when they had last eaten. There were no objective measures to control if participants complied with the instruction to fast. All participants gave informed written consent and the study was approved by the Ethics Committee of the Charité Universitätsmedizin.

### Tasks

#### Devaluation paradigm

**Figure 1:**
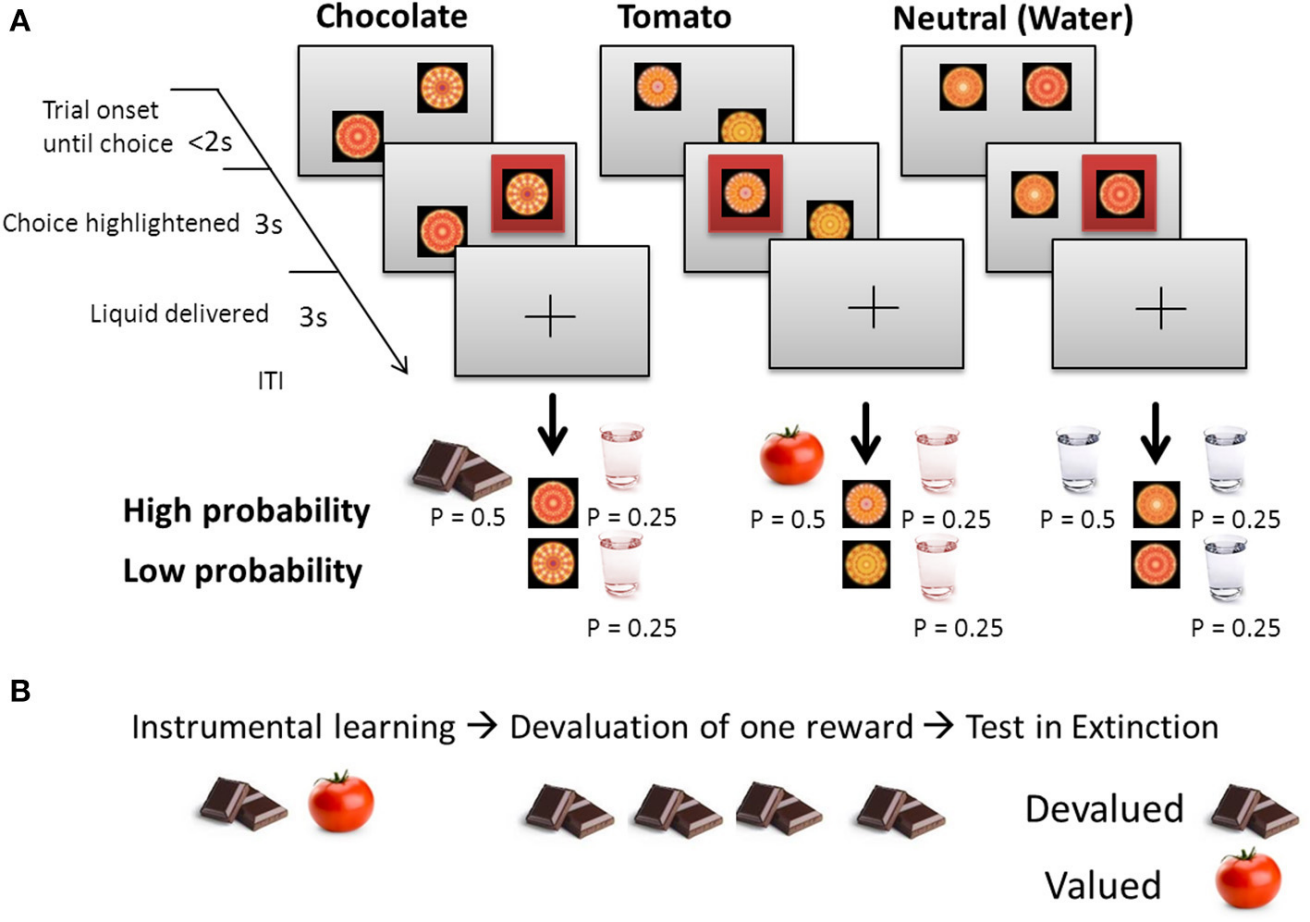
**Selective devaluation paradigm according to Valentin et al. (2007). (A)** Trial structure depicted for each condition during instrumental learning. On each trial subjects had to choose between two abstract stimuli, the chosen stimulus is then highlighted. The high-probability stimulus choice leads to a food outcome (chocolate or tomato) with a probability of *p* = 0.5 and to a common outcome (fruit tea) with *p* = 0.25. The low-probability stimulus choice never leads to food outcomes and in *p* = 0.25 to the common outcome. In the neutral condition, the high-probability stimulus choice leads to water with *p* = 0.75 and the low-probability stimulus choice to water with *p* = 0.25. **(B)** After instrumental training, subjects were invited to consume either chocolate (illustrated here) or tomato soup to satiety, resulting in a selective devaluation of the consumed outcome. Subjects then underwent the same “test” procedure in extinction (chocolate and tomato were no longer presented).

To test goal-directed vs. habitual behavior, we used a selective devaluation paradigm with liquid food rewards (Figures 1A,B; Valentin et al., 2007). The two liquid food rewards were chocolate milk and tomato juice. These foods were chosen because they can be administered in liquid form, are palatable at room temperature and are distinguishable in their flavor and texture to help facilitate sensory specific satiety effects. In addition we also used a tasteless neutral water solution and fruit tea as control. The food rewards were delivered by means of separate electronic syringe pumps (one for each liquid) positioned behind a small room divider (paravent). These pumps transferred the liquids to the subjects via plastic tubes (~6 mm diameter). The end of these tubes were held between the subject's lips like a straw and attached to the shoulder with a small adhesive tape while they were sitting in front of the computer screen performing the task.

The task consisted of three trial types: chocolate, tomato or neutral, with fully randomized order throughout the experiment (Figure 1A). On each trial, subjects were faced with the choice between two abstract stimuli, each of which was associated with different probabilities to receive a rewarding liquid food outcome or nothing.

The experimental procedure (Figures 1A,B) was divided into three steps: (1) training, (2) devaluation, and (3) test in extinction. *First*, during the *training* part, subjects learned to make choices that were associated with the subsequent delivery of these different liquid food outcomes (0.5 ml of tomato juice or chocolate milk and fruit tea). For each trial type, the overall probability of a food outcome was *p* = 0.75 for the high-probability stimulus (referring to the choice of the stimulus associated with a high-probability food outcome) with *p* = 0.5 for tomato or chocolate and *p* = 0.25 for the common outcome fruit tea. The low probability stimulus (meaning the choice of the stimulus associated with a low probability liquid food outcome) led with *p* = 0.25 to a common outcome (0.5 ml fruit tea). In the control condition, water was delivered with the same probabilities of *p* = 0.75 after a high probability stimulus choice and *p* = 0.25 after a low probability stimulus choice, respectively. The training sessions consisted of 150 trials (50 trials for each stimulus pair). To facilitate learning of the stimulus-outcome associations between the abstract stimuli and the liquid food rewards, each stimulus-outcome pair (chocolate, tomato, and neutral) was randomly assigned to one of the four spatial positions on the screen (top left, top right, bottom left, or bottom right) at the beginning of the experiment and remained constant throughout. A unique spatial location was assigned to the high-probability stimulus in all three trial-type pairs. The specific assignment of arbitrary fractal stimuli and spatial position to each particular action was fully counterbalanced across subjects. The subjects' task on each trial throughout all parts of the experimental procedure was to choose one of the two possible available stimuli on the screen which they perceived as being “more pleasant” (and thus is associated with a higher probability to receive a rewarding outcome). In a *second* step, during the *devaluation* part and after training, either tomato juice or chocolate milk was selectively devalued by feeding the subject with the food until they reported a feeling of satiety. For devaluation, participants ate either chocolate pudding or tomato soup (mean consumption in grams = 357.1, std. = 196.0) until they rated the devalued food as unpleasant and refused to consume more. *Third*, during *test in extinction* and after devaluation, participants continued with the instrumental choice paradigm in extinction without delivery of the liquid food rewards tomato or chocolate (150 trials without food delivery, 50 trials for each stimulus pair). To maintain some degree of responding, the neutral fruit tea outcome continued to be available as during training with equal probability for the two available actions of *p* = 0.3 each (similar to Valentin et al., 2007). Subjects rated pleasantness of all administered foods on a visual analogous scale (VAS) before training, after training, after devaluation and after extinction. The use of an extinction procedure ensured that subjects only use information about the value of the outcome by making use of the previously learned associations between that outcome and a particular choice, as otherwise, if the tomato and chocolate outcome were presented again at test, subjects could relearn a new association, thereby confounding stimulus-response and response-outcome contributions. As reported by Valentin et al. (2007), goal-directed behavior is characterized by a significant decrease in choices of the stimulus associated with the devalued outcome, whereas habitual behavior leads to continued choosing of the stimulus associated with the devalued outcome. The number of choices was analyzed using a 2 (time: pre/post) × 2 (value: devalued/valued) repeated measures ANOVA to assess the degree of goal-directed vs. habitual choices.

For the devaluation paradigm, four participants had to be excluded from the sample (2 did not reach the learning criterion of 75% correct choices during training session and 2 refused to eat more although they did not rate the devalued food as being less pleasant after consumption and thus did not reach satiety).

#### Sequential decision-making task

**Figure 2:**
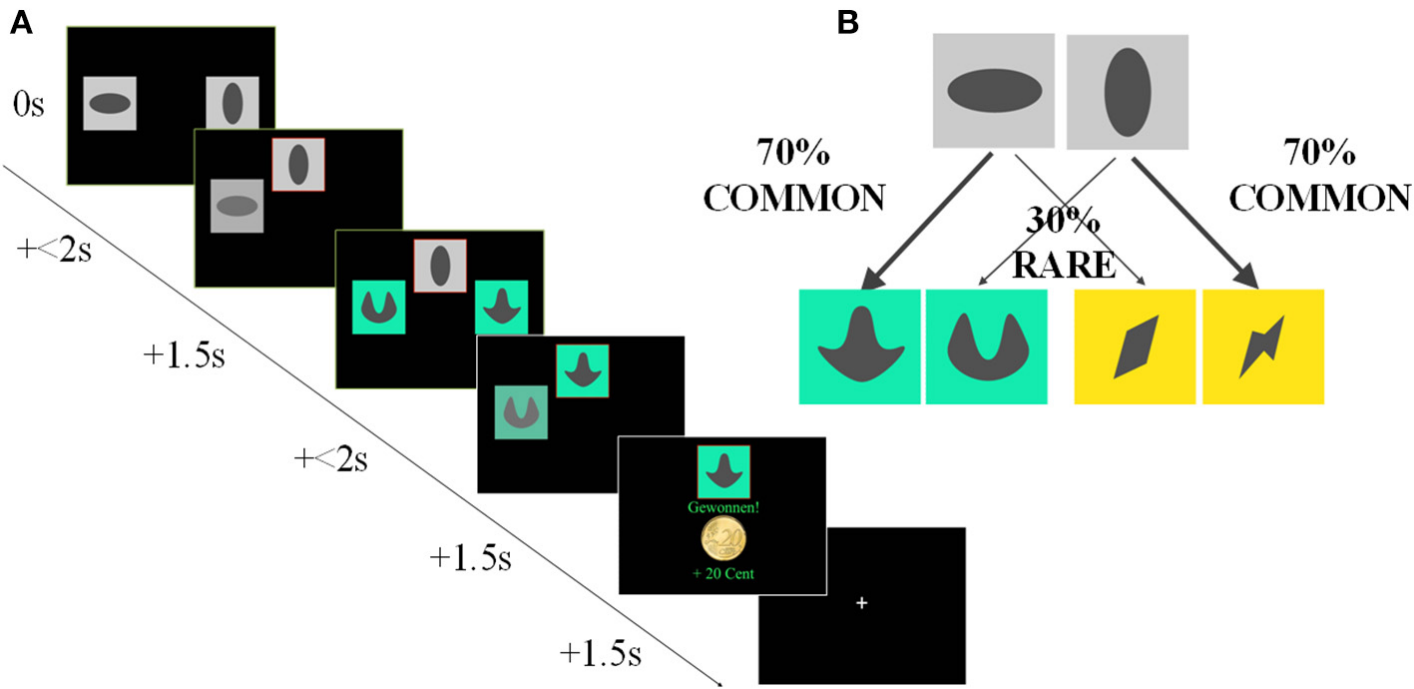
**Sequential Decision-Making Task (Two-Step), according to Daw et al. (2011). (A)** Trial configuration for the Experiment. Each trial consisted of two different stages, and each stage involved a choice between two stimuli. In the first stage, subjects chose between two abstract stimuli on a gray background. The chosen stimulus was highlighted by a red frame and moved to the top of the screen, where it remained visible for 1.5 s; at the same time, the other stimulus faded away. Subjects then reached a subsequent second stage. Here subjects saw one of two further pairs of colored stimuli and again chose between these. The monetary outcome following this second stage choice (gain or no gain of 20 cent) was then presented centrally on the screen. **(B)** One pair of colored second stage stimuli occurred commonly (on 70% of trials; “common trials”) after choice of one first stage stimulus, while the other pair was associated equally strongly with the other stimulus. On the remaining 30% of trials, the chosen first stage option resulted in a transition to the other second stage stimulus pair (“rare trials”). Reinforcement probabilities for each second stage stimulus changed slowly and independently according to Gaussian random walks with reflecting boundaries at 0.25 and 0.75.

In the sequential decision-making task (Daw et al., 2011), participants had to make two subsequent choices (each out of two options) to finally receive a monetary reward. At the first stage, each choice option led commonly (with 70% probability) to one of two pairs of stimuli and rarely (with 30% probability) to the other one. After entering the second stage, a second choice was followed by monetary reward or not, which was delivered according to slowly changing Gaussian random walks to facilitate the continuous updating of the second-stage action values. Participants performed a total of 201 trials. Crucially, a purely model-based learner uses the probabilities that underlie the transition from the first to the second stage, while a purely model-free learner neglects this task structure (Figure 2). Depending on the impact of previous second-stage rewards on the following first-stage choices, reinforcement learning theory predicts distinct first-stage choice patterns for model-free as opposed to model-based strategies. Model-free behavior can only generate a main effect of reward: a rewarded choice is more likely to be repeated, regardless of whether the reward followed a common or rare transition. Model-based behavior results in an interaction of the two factors, because a rare transition inverts the effect of the subsequent reward. Stay-switch behavior was analyzed as a function of reward (reward/no reward) and state (common/rare). These individual stay probabilities were subjected to a repeated-measures ANOVA with the factors reward and state.

With respect to the sequential decision-making task, one participant was excluded due to abortion of the experiment after half of the trials.

### Computational modeling of the sequential decision-making task

The aim of model-free and model-based algorithms is to learn values for each of the stimuli, which appear in the task as three pairs (*s*_*A*_, *s*_*B*_, *s*_*C*_). *s*_*A*_ refers to the first-stage stimuli and *s*_*B*_ and *s*_*C*_ to the two pairs of second-stage stimuli. Here, *a* refers to the chosen stimuli and the indices *i* and *t* denote the stage (*i* = 1 for *S*_*A*_ at the first stage and *i* = 2 for *S*_*B*_ or *S*_*C*_ at the second stage) and the trial, respectively. The model-free algorithm was SARSA(λ):

(1) $$Q_{MF_{s_{i,t+1},a_{i,t+1}}}\;=\;Q_{MF_{s_{i,t},a_{i,t}}}+\;\alpha_{i}\delta_{i,t}$$

(2) $$\delta_{i,t}\;=r_{i,t}+\;Q_{MF_{s_{i+1,t},a_{i+1,t}}}-\;Q_{MF_{s_{i,t},a_{i,t}}}$$

Notably, *r*(*s*_1, *t*_) = 0 because there are no rewards available at the first stage and Q(*s*_3, *t*,_ *a*_*t*_) = 0 at the second stage because there are only two-stages and no third stage in this version of a sequential decision making task. All Q-values were initialized (“starting parameter”) with 0. We allow different learning rates α_*i*_ for each stage *i.* Further, we allow for an additional stage-skipping update of first-stage values by introducing another parameter λ, which connects the two stages and allows the reward prediction error at the second stage to influence first-stage values:

(3) $$Q_{MF_{s_{1,t+1},a_{1,t+1}}}=Q_{MF_{s_{1,t},a_{1,t}}}+\;\alpha_{1}\lambda \delta_{2,t}$$

It is worth mentioning that λ additionally accounts for the main effect of reward as observed in the analysis of first-stage stay-switch behavior but not for an interaction of reward and state. Instead, the introduction of the transition matrix accounts for this interaction. Here, the model-based algorithm learns values by taking into account the transition matrix and computes first-stage values by simply multiplying the better option at the second stage with the transition probabilities:

(4) $$\begin{array}{l} Q_{MB}{}_{s_{A},a}=P(S_{B}|S_{A},a)\;\times \;\max Q_{MF}{}_{s_{B},a}+P(S_{c}|S_{A},a)\\ \;\times \;\max Q_{MF}{}_{s_{C},a} \end{array}$$

(5) $$Q_{MB_{s_{2,t},a_{2,t}}}\;=Q_{MF_{s_{2,t},a_{2,t}}}$$

Note that this approach simplifies transition learning because transition probabilities are not learned explicitly. This approach is in line with the task instructions, and a simulation by Daw et al. (2011) verified that this approach outperforms incremental learning of the transition matrix (compare Wunderlich et al., 2012 but also see Glascher et al., 2010 or Lee et al., 2014). Finally, we connect *Q*_*MF*_ and *Q*_*MB*_ in a hybrid algorithm:

(6) $$Q_{s_{A},a}\;=\omega \;\times \;Q_{MB_{s_{A},a}}+\;\left(1-\omega \right)\;\times \;Q_{MF_{s_{A},a}}$$

(7) $$Q_{s_{2},a}\;=Q_{MB_{s_{2},a}}=Q_{MF_{s_{2},a}}$$

Importantly, ω gives a weighting of the relative influence of model-free and model-based values and is therefore the model's parameter of most interest. To generate choices, we apply a softmax for *Q*:

(8) $$p(i,a,t)=\;\frac{\exp\;(\beta_{i}(Q_{s_{i,t,{a^{\prime }}_{i,t}}}+\rho \;\times \;rep(a)))}{\sum_{a^{\prime }}\exp\;(\beta_{i}(Q_{s_{i,t},{a^{\prime }}_{i,t}}+\;\rho \;\times \;rep(a^{\prime })))}$$

Here, β controls the stochasticity of the choices and we assume this to be different between the two stages. The additional parameter ρ captures first-stage choice perseveration and rep is an indicator function that equals 1 if the previous first-stage choice was the same (Lau and Glimcher, 2005; Daw et al., 2011). In summary, the algorithm has a total of 7 parameters and can be reduced to its special cases ω = 1 (4 parameters) and ω = 0 (5 parameters). We fit bounded parameters by transforming them to a logistic (α, λ, ω) or exponential (β) distribution to render normally distributed parameter estimates. To infer the maximum a posteriori estimate of each parameter for each subject, we set the prior distribution to the maximum-likelihood given the data of all participants and then use Expectation-Maximization. For an in-depth description please compare Huys et al. (2011) and Huys et al. (2012). In the computational modeling part there were no differences to Daw et al. (2011) with respect to the applied model-free and model-based algorithms as well as the softmax function.

### Correlation analysis of goal-directed and model-based behavior

We assessed the degree of goal-directed behavior in the selective devaluation task by computing the interaction score from the number of choices: *“(valued stimulus pre devaluation – valued stimulus post devaluation) – (devalued stimulus pre devaluation - devalued stimulus post devaluation).*” Here, a higher score indicates more goal-directed behavior, i.e., participants more frequently preferred the valued over the devalued stimulus after devaluation. Model-based behavior in the sequential decision-making task was assessed with a similar interaction score of stay probabilities at the first stage: “*(rewarded common stimulus choice – rewarded rare stimulus choice) – (unrewarded common stimulus choice - unrewarded rares stimulus choice)*.” This indicates more model-based behavior when participants more frequently stayed after having received rewards in common states and no-rewards in rare states. Further, we also use the parameter ω derived from computational modeling which balances the influences of model-free and model-based decision values. Based on a directed a priori hypothesis of a positive association between the main outcome measures of the two paradigms, we report one-tailed *p*-values. Due to the relatively small sample size, we apply the more conservative Spearman correlation coefficient, which is more robust against outliers.

## Results

### Devaluation paradigm

#### Training

**Figure 3:**
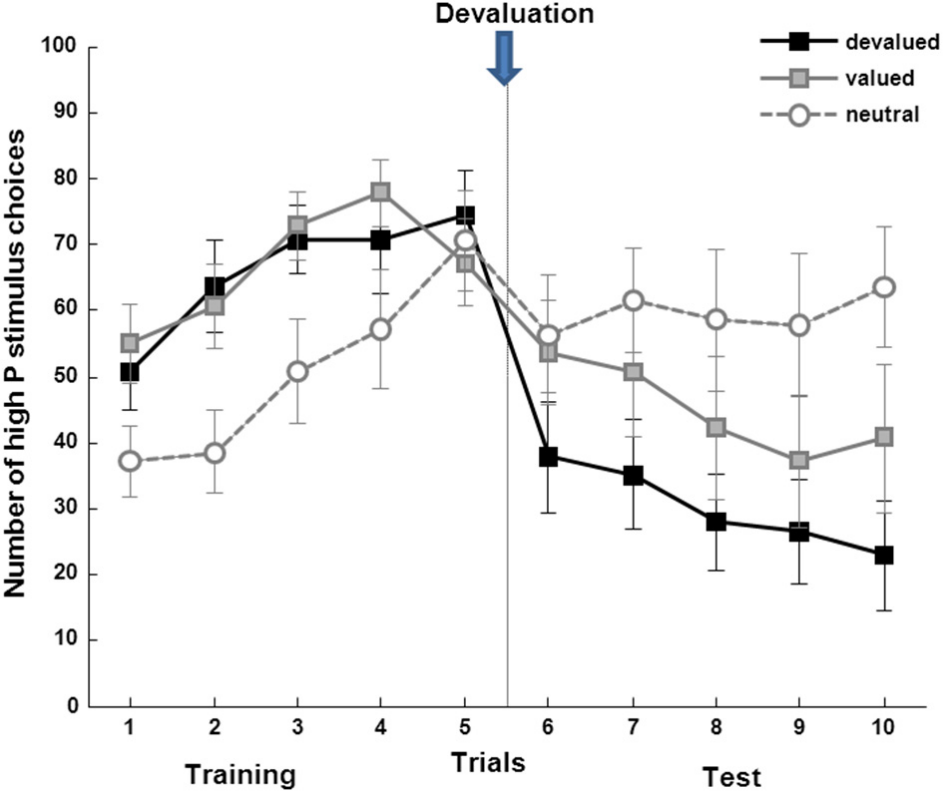
**Subjects learn to choose the high probability stimulus in all three conditions (devalued: *T* = −3.50, *p* = 0.004, valued: *T* = −2.60, *p* = 0.022; and neutral: *T* = −2.73, *p* = 0.017), with a significant decrease of instrumental choice after devaluation only for the devalued stimulus (*T* = 3.15, *p* = 0.008)**.

Over the course of training, participants (*n* = 14) chose the high-probability stimulus (delivering the rewarding food with a higher probability) significantly more often compared to the low probability stimulus (Figure 3). This was the case for all stimuli associated with the high-probability outcome in the last 10 trials of the training session [the devalued (*T* = −3.50, *p* = 0.004), valued (*T* = −2.60, *p* = 0.022), and neutral (*T* = −2.73, *p* = 0.017) condition].

#### Outcome devaluation

**Figure 4:**
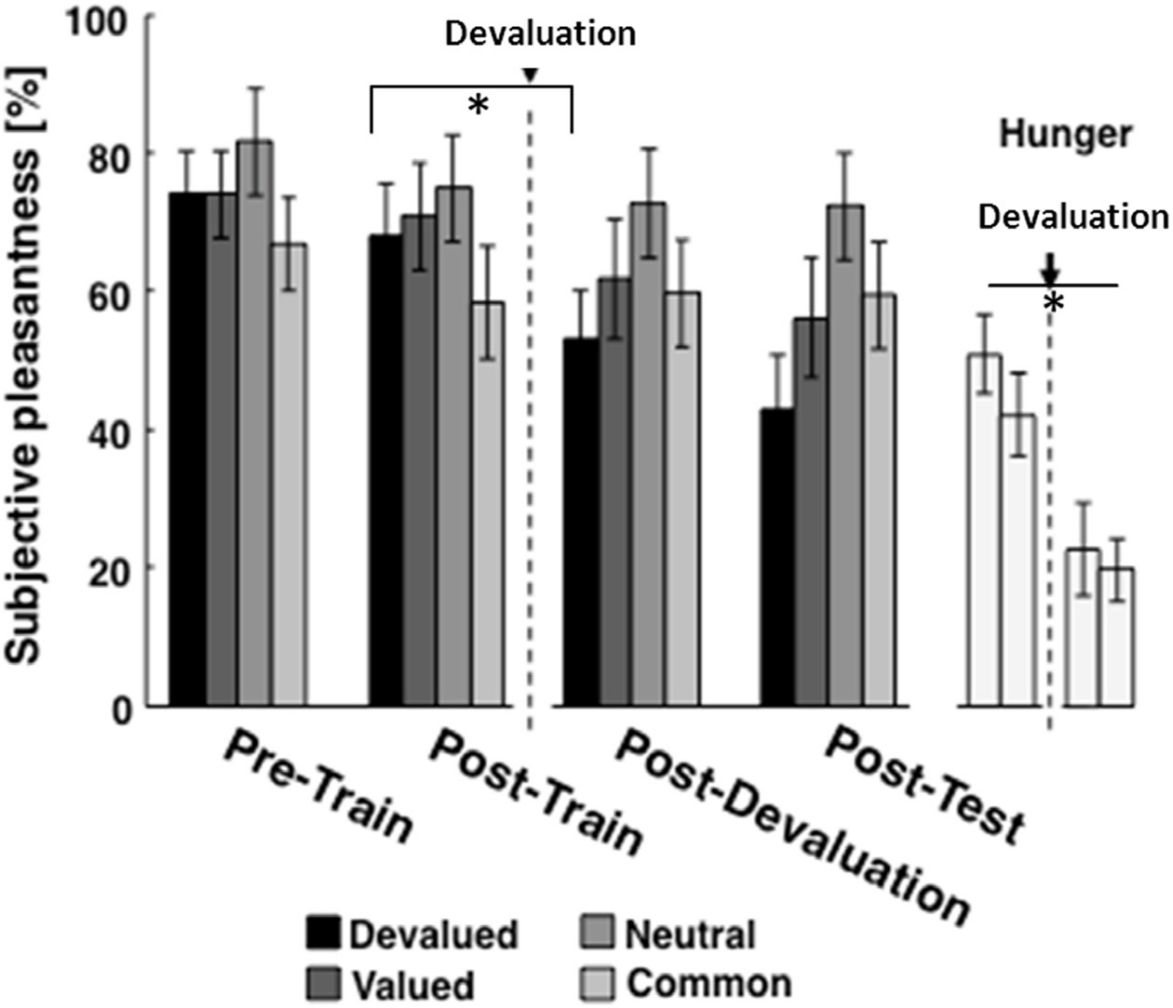
**Subjective pleasantness ratings for the devalued (chocolate or tomato), the neutral (water), and the common (fruit tea) outcome at 4 time points throughout the experimental procedure**. After devaluation, participants rated the devalued food (chocolate or tomato) significantly (as indicated by ^*^) less pleasant compared to the valued and neutral condition (*T* = 2.67, *p* = 0.019). Further, they reported significantly less hunger after the devaluation procedure (panels display subjective hunger ratings at the 4 time points, *T* = 2.25, *p* = 0.042).

After devaluation, participants rated the devalued food (chocolate or tomato) significantly less pleasant compared to the valued and neutral condition (Figure 4, *T* = 2.67, *p* = 0.019). Further, they reported significantly less hunger after the devaluation procedure (Figure 4, *T* = 2.25, *p* = 0.042). These results clearly indicate that the devaluation exerted its expected effect selectively for the devalued but not for the valued outcome.

#### Test phase in extinction

**Figure 5:**
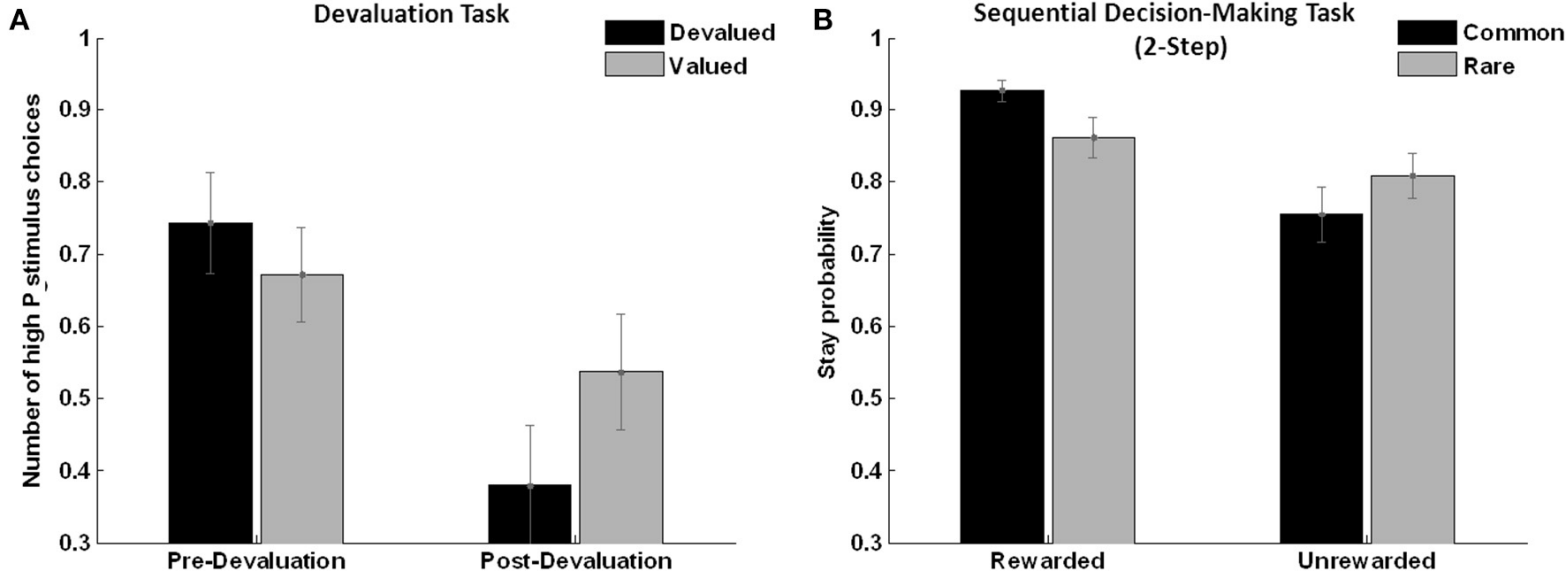
**Analysis of choice behavior. (A)** Devaluation task: subjects show significantly more valued compared to devalued stimulus choices after devaluation in extinction (*n* = 14, *F* = 5.20, *p* = 0.040, error bars indicate s.e.m.), reflecting “goal-directed” behavior. **(B)** Sequential choice task: the same subjects show higher stay probabilities in the “rewarded common” as opposed to the “unrewarded common” trials (main effect reward: (*n* = 17, *F* = 14.1, *p* = 0.002), reflecting “model-based” behavior with a positive reward × frequency interaction over all subjects (*n* = 17, *F* = 6.05, *p* = 0.026).

Assessing choice behavior after the devaluation procedure during the test phase in extinction, a significant time (pre/post training) × condition (devalued/valued/neutral) interaction was found (*F* = 5.200, *p* = 0.040, see Figure 5). This was due to a significant decrease in choice of the high-probability stimulus associated with the devalued compared to the stimulus associated with the valued and neutral outcome in the first 10 trials of the test session compared to the last 10 trials of the training session (*T* = 3.15, *p* = 0.008). Thus, participants were able to adapt their choices of stimuli as a function of the associated outcome value, providing direct behavioral evidence for goal-directed behavior as has been previously reported by Valentin et al. (2007).

### Sequential decision-making task (two-step)

In line with previous studies (Daw et al., 2011; Wunderlich et al., 2012; Smittenaar et al., 2013), stay-switch behavior at the first stage revealed a significant main effect of reward (*F* = 14.1, *p* = 0.002) and a significant reward × state interaction (*F* = 6.05, *p* = 0.026, see Figure 5). This clearly shows that both rewards and state transitions influenced the participants' choices. Thus, a mixture of model-free and model-based strategies was observed and this was further quantified using a computational model that weights the influence of both strategies. Distribution of random-effects parameters from computational modeling is displayed in Table 1.

**Table 1:** **Best-fitting parameter estimates shown as median plus quartiles across subjects**.

	**β1**	**β2**	**α1**	**α2**	**λ**	**ω**	**p**
25th percentile	4.57	1.53	0.28	0.40	0.44	0.34	0.15
median	6.55	2.42	0.56	0.58	0.70	0.43	0.20
75th percentile	7.55	4.35	0.76	0.69	0.85	0.54	0.26

β, stochasticity of the choices for the first (β1) and second (β2) stage; α, learning rate for first (α1) and second (α2) stage; λ, reinforcement eligibility parameter (estimated value of the second stage should act as the same sort of model-free reinforcer for the first stage choice); ω, relative influence of model-free and model-based values; p, first-stage choice perseveration.

### Construct validity: correlation between both measurements

**Figure 6:**
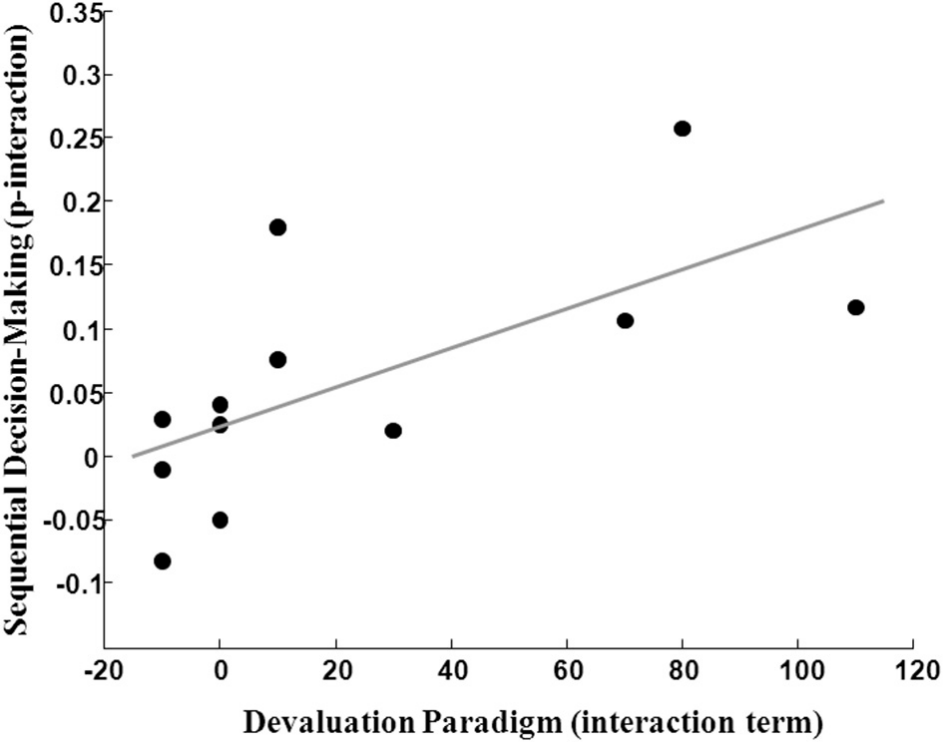
**Correlation of “model-based” [sequential decision-making (2-step) interaction term] and “goal-directed” (devaluation paradigm interaction term) behavior (*n* = 12, Spearman *R* = 0.74, *p* = 0.003)**.

Thirteen subjects were included in the final analysis of both tasks (mean age in years = 46, std = 9). The interaction score derived from the outcome devaluation task correlated significantly with the interaction score derived from the sequential decision-making task (Spearman's rho = 0.708, *p* < 0.005, one tailed) and also with the parameter ω derived from computational modeling (Spearman's rho = 0.498, *p* < 0.05, one tailed). When removing one outlier for the model-based score (3*SD* > mean), the correlation still remained significant in 12 participants (Figure 6).

Interestingly, the interaction term from the selective devaluation task did not correlate with the main effect of reward or with the parameter λ (scaling the influence of reward prediction errors on first-stage decision values) derived from computational modeling (*p* > 0.75).

## Discussion

In the present study, we used two reinforcement learning tasks in the same participants, selective devaluation and sequential decision-making, which are frequently used in human research. Here, we aim to assess the construct validity of these two measurements which have both been suggested to capture the dichotomy of goal-directed or model-based vs. habitual or model-free control, respectively. In the selective devaluation task, we found evidence of goal-directed choices as subjects decreased their choice for a stimulus associated with a now devalued outcome. In the sequential decision-making task, subjects displayed model-based behavior, which is by definition goal-directed, indicating that participants used the transition structure to solve the task as it is indicated by the significant reward by state interaction and by the weighting parameter ω derived from computational modeling.

As comprehensively reviewed by Dolan and Dayan (2013) those two different operationalizations in part stem from different methodological and historical perspectives. Both selective devaluation and sequential decision-making have been used to describe similar behavioral patterns but they have never been directly related to one another in a sample of human subjects. Here we found, that measures of the individual degree of goal-directed behavior assessed with selective devaluation and model-based behavior assessed during sequential decision-making indeed correlate positively. This provides evidence for the construct validity of both measurements indicating that they measure the same concept grounded in a single common framework as suggested by Dolan and Dayan (2013).

Here, we suggest that goal-directed behavior as measured during selective devaluation reflects one of the many facets of model-based learning which is also applicable to several other tasks, in particular instrumental reversal learning (Hampton et al., 2006; Li and Daw, 2011; Schlagenhauf et al., 2014) but also Pavlovian conditioning (Huys et al., 2012; Prevost et al., 2013). This may suggest that the individual balance between the two different modes of control over instrumental choices may be relevant for a variety of tasks and reflect enduring interindividual differences that are consistent across tasks. Although this balance between goal-directed and habitual control has been considered as interindividual trait (Doll et al., 2012; Dolan and Dayan, 2013) we have to caution that the temporal stability of these measures has not been shown—as it has been the case e.g., for cognitive functions like working memory (Klein and Fiss, 1999; Waters and Caplan, 2003).

Another related question—not addressed here—concerns the notion by Daw et al. (2005) that model-free and model-based learning strategies compete with each other based on the relative certainty of their estimates (Daw et al., 2005). From this theoretical perspective, the model-based system is computationally costly: When individuals face a decision problem, the costs of opportunities of the model-based system need to rule out the benefits of the simple model-free system to govern control over a decision (also compare Niv et al., 2007). In other words, use of the model-based system should be beneficial compared to the model-free system. Lee et al. (2014) suggested that an arbitrator keeps track of the degree of reliability of the two systems and uses this information in order to proportionately allocate behavior control depending on task demands.

The sequential decision-making task used in the present study gives an individual degree of both model-free and model-based behavior. We observed that the degree of goal-directed behavior in the devaluation task was not related to measurements representing the degree of the model-free behavior during sequential decisions (as indicated by the main effect of reward or a high reinforcement eligibility parameter derived from computational modeling). This indicates the specificity of the correlation of goal-directed choices measured with the devaluation procedure and the degree of model-based behavior measured with the sequential decision-making task. One might have expected that a continued choice of the devalued option indicates habitual behavior which is then represented in a small interaction term. A correlation of the interaction term of the devaluation paradigm with measures of model-free behavior in the sequential decision-making task would have indicated that habitual behavior can be induced by the devaluation procedure. The absence of such an association is in line with the findings from Valentin et al. (2007) that on the neuronal level no activation of structures associated with habitual behavior like e.g., the putamen was observed so that the authors conclude that their selective devaluation paradigm is indeed better suited to reflect goal-directed behavior whereas habitual behavior might be observed in tasks using overtraining (Tricomi et al., 2009). To this end, associations between the balance in between model-based and model-free control determined in sequential decision-making should be related to behavioral measures of habitual responding in overtraining paradigms. In the sequential decision-making task used here the outcome probabilities driving model-free behavior during sequential decision-making were changing slowly (according to Gaussian random walks) to facilitate continuous updating of decision values. This was implemented to avoid a moment in time during the task when a purely model-free strategy becomes clearly advantageous compared to the more complex model-based strategy and might have had an effect on the development of habit-like patterns.

Thus, it is important to note that both paradigms may provide different insight into the habitual system, while goal-directed/model-based measurements are more related (and can be better captured via the two experimental procedures). For example in another variant of devaluation (De Wit et al., 2009) alcohol-dependent patients indeed displayed an overreliance on habits at the cost of goal-directed behavior (Sjoerds et al., 2013). Using sequential decision-making in alcohol-dependent patients, another study demonstrated that model-based behavior is compromised but no difference between patients and controls was observed in terms of model-free behavior (Sebold et al., 2014). While sequential decision-making enables researchers to disentangle model-free and model-based contributions to decision-making, it may obscure enhanced habit-like patterns. To this end, paradigms are needed that are rigorously designed to capture the appropriate predominance of one or the other mode of control given a certain moment in time, also taking into account an arbitrator evaluating the performance of each of these systems (as described by Lee et al., 2014).

Limitations of our study include a relatively small sample size, thus both paradigms and the assessed measurements have been previously validated separately in larger samples (Valentin et al., 2007; Daw et al., 2011; Wunderlich et al., 2012). All results are correlational, hence inferences about causality are very limited. Nevertheless, the strong a priori hypothesis of one, single framework supports the idea of construct validity as assessed by the reported correlation.

Summing up, we suggest that the same construct of goal-directed and model-based behavior is assessed via different experimental procedures (devaluation and sequential decision-making) that validly measure this construct. This is the first study to directly compare these experiments in one sample of human participants. In conclusion, our results support the longstanding and pervasive idea of a common single framework. Therefore, we provide evidence for the construct validity, which merits the use of both experiments in assessing interindividual differences in the predominant type of behavioral control over instrumental choices.

### Conflict of interest statement

The authors declare that the research was conducted in the absence of any commercial or financial relationships that could be construed as a potential conflict of interest.
